# Renal diseases associated with multiple sclerosis: A narrative review

**DOI:** 10.1097/MD.0000000000031959

**Published:** 2022-12-02

**Authors:** Gabriel Alugba, Alexsandra Urhi, Iyanu Victoria Olateju, Henry Onyemarin, Consolata Uzzi, Tolulope Oshiba-Fowode, Elvis Obomanu, Hakeem Adegboyega Popoola, Emeka J. Okoronkwo, Emmanuel Ukenenye, Gideon Asaolu, Adeolu Funso Oladunjoye, Olubunmi Oladunjoye

**Affiliations:** a Delta State University, Abraka, Nigeria; b Federal Neuropsychiatric Hospital, Benin City, Nigeria; c Department of Internal Medicine, Medstar Union Memorial Hospital, Baltimore, MD, USA; d Asaba Specialist Hospital, Asaba, Delta State, Nigeria; e Columbus Specialty Hospital, Newark, NJ, USA; f Department of Internal Medicine, University of Texas Health Science Center, TX, USA; g Department of Medicine, University of Portharcourt, Choba, Nigeria; h Windsor University School of Medicine, Cayon, St. Kitts; i Lagos University Teaching Hospital, Nigeria; j Medical Council of Jamaica, University of the West Indies, Kingston, Jamaica; k Menninger Department of Psychiatry, Baylor College of Medicine, Houston, TX, USA; l Department of General Internal Medicine, Baylor College of Medicine, Houston, TX, USA.

**Keywords:** multiple sclerosis, renal diseases

## Abstract

The mechanisms of renal pathology in multiple sclerosis (MS) can be related to the disease itself or its treatment. Although kidney disease can be associated with MS, not much has been reported in the literature; hence, our study aimed to describe the prevalence and types of renal diseases and discuss their prognosis in patients with MS. A literature search (2012–2022) was performed using the Scale for the Assessment of Narrative Review Articles. The databases searched included MEDLINE (PubMed) and EMBASE. Fourteen articles from these databases met the inclusion criteria. The inclusion criteria were as follows: publications with full-text access. Articles published in English. Original articles related to renal diseases in MS. The prevalence of renal diseases in MS from the articles obtained ranged from 0.74% to 2.49%. Interferon beta (IFN-β)-associated glomerulonephritis was common among the reviewed articles. Significant improvement and resolution of the pathology were observed after the discontinuation of the offending medication. Renal symptoms in 2 out of 4 cases with renal thrombotic microangiopathy (TMA) induced by interferon-beta progressed to chronic kidney disease, even after the drug was stopped. Other studied renal pathologies included nephrolithiasis secondary to urinary retention and urinary catheter use in patients with MS.

## 1. Introduction

Multiple sclerosis (MS) is a demyelinating inflammatory disease of the central nervous system,^[1]^ it is one of the leading causes of progressive neurological disability in young adults^[2]^ with an estimated prevalence of 85 per 100,000 in the United States and 100 per 100,000 in Europe.^[3,4]^ It has historically been classified as an organ-specific T-cell mediated autoimmune disease. However, the success of B-cell targeted therapies challenges the standard T cell autoimmune dogma. It is traditionally viewed as a 2-stage disease, with early inflammation responsible for relapsing-remitting disease and delayed neurodegeneration causing non-relapsing progression, that is, secondary and primary progressive MsS.^[5]^

Common manifestations include weakness, spasticity, visual disturbances, sensory defects, cognitive problems and depression.^[6]^ Genitourinary symptoms are common and have been reported in about 90% of MS patients, with neurogenic vesicourethral dysfunction being a common manifestation of MS.^[7,8]^ It has been one of the leading socially-disabling sequelae of MS.^[7]^ Urodynamic abnormalities such as neurogenic detrusor over activity, detrusor sphincter dyssynergia, and detrusor hypocontractility have been reported in up to 99%, 83%, and 40% of these patients, respectively.^[9]^ The upper urinary tract abnormalities in MS which have been seen in 0% to 25% of patients include pyelonephritis, hydronephrosis, vesicoureteral reflux, and nephrolithiasis.^[9]^

There is a paucity of data on renal parenchymal diseases associated with MS, this may be due to the rarity of kidney pathology in MS, and also may be due to the resolution of symptoms when treatment is commenced. Available studies indicate that most causes of renal disease are related to the adverse effects of treatment and sequelae from lower urinary tract dysfunction in patients with MS. Despite the fact that MS affects multiple organ systems, its role in renal pathology has received little attention. This review aimed to describe the prevalence of associated renal diseases, types of renal diseases, and discuss the prognosis of renal diseases in patients with MS.

## 2. Methodology

### 2.1. Search strategy

We searched the literature for relevant studies, including case reports, case series, systematic reviews, and clinical trials on MS-associated renal diseases from 2012 to 2022. The databases used included MEDLINE (PubMed) and EMBASE. The search terms used were “*renal disease*,” “*kidney disease*,” and “*multiple sclerosis*”, which were combined using the Boolean operator “AND.” A preliminary search was carried out and verified by 2 authors (GA and AU). All identified articles were uploaded to Abstrackr (abstract screening software). All the authors screened the articles based on the inclusion and exclusion criteria. Following the initial screening of abstracts, the full text of each article was reviewed, and 12 articles that met the inclusion criteria were included. All conflicts regarding article selection were resolved through 2-team discussions.

### 2.2. Inclusion and exclusion criteria

Our inclusion criteria were publications with full-text access indexed in PubMed and EMBASE, articles published in English, and original articles related to renal diseases in MS. Our exclusion criteria included articles published in languages other than English; articles irrelevant to renal diseases in MS; and all narrative reviews, systematic reviews, meta-analyses, and conference materials.

## 3. Result

Ten of twelve of the articles were case reports, one was a case series, and one was a retrospective study (Tables 1 and 2). The prevalence of renal disease in MS ranged from 0.74% to 2.49%.^[21]^ The most common type of renal disease reported is glomerulonephritis,^[11–14,16,17]^ which is induced by IFN-β therapy. Significant improvement in symptoms and resolution of pathology were observed when the offending medication was removed. Renal thrombotic microangiopathy (TMA) was the next most prevalent renal disease identified and was secondary to IFN-β treatment.^[15,18–20]^ However, 2 cases progressed to chronic kidney disease even after IFN β was discontinued.^[15,19]^ Other renal pathologies included acute renal failure from nephrogenic lower urinary tract dysfunction,^[10]^ and nephrolithiasis^[1]^ found among MS patients.

**Table 1:** Summary of findings from case reports and series used for this narrative review.

Authors	Age	Sex	Diagnosis	Identified risk factor	Biopsy results	Treatment	Dialysis/Renal transplant	Outcome
1. Yuruktumen et al, 2004^[10]^	64	M	Acute renal failure	Neurogenic lower urinary tract dysfunction	NA	Urinary catheterization	No	Resolved
2. Tornes et al, 2012^[11]^	41	F	Glomerulonephritis	IFN β-1a	FSGS	Stopping the offending drug	No	Resolved
3. Ozturk et al, 2016^[12]^	32	M	Glomerulonephritis	IFN β-1a	FSGS	Stopping the offending drug	No	Resolved
4. Auty and Seleh, 2005^[13]^	28	M	Glomerulonephritis	IFN β-1a	MG	Stopping the offending drug	No	Resolved
5. Wallbach et al, 2013^[14]^	40	F	Glomerulonephritis	IFN β-1a	MPGN	Replacing IFN-1b with glatiramer acetate	No	Resolved
6. Mahe et al, 2013^[15]^	38	F	Renal TMA	IFN β-1a	TMA	Stopping the offending drug	No	Progressed
7. Kumasaka et al, 2006^[16]^	43	F	Glomerulonephritis	IFN β-1b	MCD	Stopping the offending drug	No	Resolved
8. *Markowitz et al, 2010^[17]^	27	F	Glomerulonephritis	IFN β-1a	FSGS	Stopping the offending drug	No	Unknown
*Markowitz et al, 2010^[17]^	33	F	Glomerulonephritis	IFN β-1b	FSGS	Stopping the offending drug	No	Resolved
*Markowitz et al, 2010^[17]^	37	F	Glomerulonephritis	IFN β- 1a	FSGS	Stopping the offending drug	No	Resolved
9. Omoto et al, 2018^[18]^	42	F	Renal TMA	IFN β- 1b	TMA	Stopping the offending drug	No	Resolved
10. Broughton et al, 2011^[19]^	53	F	Renal TMA	IFN β-1b	TMA	Stopping the offending drug	No	Progressed
11. Baghbanian and Moghadasi et al, 2018^[20]^	38	F	Renal TMA	IFN β-1a	TMA	Stopping the offending drug	No	Resolved

F *=* female, FSGS *=* focal segmental glomerulosclerosis, IFN β-1a *=* interferon β-1a, IFN β-1b *=* interferon β-1b, M *=* male, MCD = minimal change disease, MG *=* membranous glomerulonephritis, MPGN *=* membranoproliferative glomerulonephritis, NA *=* not available, TMA *=* thrombotic microangiopathy.

*indicating case series.

**Table 2:** Summary of findings from the retrospective study used for this review.

Author	Study population/Sample size	Renal disease implicated	Summary
Ganesan et al, 2017^[1]^	Patients at a large, urban academic tertiary care center and its satellite facilities/118 MS patients with stone disease. Out of these 118, 61 MS patients had a complete 24-h urinary stone panel analysis.	Nephrolithiasis	Compared to the control group, patients with MS were significantly more likely to have calcium phosphate stones (42% vs 15%, *P* < .001) and struvite stones (8% vs 3%, *P* = .03) and less likely to have calcium oxalate monohydrate stones (39% vs 64%, *P* < .001). -Use of intermittent straight catheterization (OR, 3.50 [95% CI, 1.89–6.47]; *P* = < 0.001) or an indwelling catheter (OR, 9.78 [95% CI, 4.81–19.88]; *P* = <0.001) for bladder emptying was significantly associated with stone disease.

CI = confidence interval, MS = Multiple sclerosis, p = *P*-value, vs = versus.

## 4. Discussion

Renal disease associated with MS has been reported in some studies, although uncommon. A study by Franz et al suggested MS patients with urodynamic abnormalities (such as spastic bladder, detrusor hypocontractility, and bladder outlet obstruction) can develop acute renal failure and if not properly managed, can lead to chronic kidney disease.^[22]^ Multiple hypotheses have suggested possible mechanisms and explanations for the incidence of renal diseases in MS patients but they still remain postulations without specific scientific proof or evidence.^[16,17,23–25]^

On the contrary, a study by Lawrenson et al showed no increased risk of renal failure in MS patients with.^[26]^ Their study reported that the rate ratio of renal failure compared with the general population for MS ranged between 0.4 and 1.3 in males and 0.5 and 2.2 in females, showing no significant increase in renal failure in patients with MS.^[26]^ Of note, renal diseases such as focal segmental glomerulosclerosis (FSGS) and immune complex-mediated membranoproliferative lupus nephritis have been reported in some patients with MS. Some studies have shown a causal relationship between those treated with IFN-β and incidence of FSGS.^[27–29]^ The incidence of FSGS in these patients cannot be directly attributed to MS, but rather to the use of interferon-gamma, whose side effects are dependent on dose, treatment duration, and presence of comorbidities such as diabetes mellitus and hypertension.^[11]^

### 4.1. Prevalence of renal diseases associated with MS

The prevalence of renal disease associated with MS is unclear and remains under-researched. A study conducted by Marrie et al reported that the prevalence of renal disease (without renal failure) in MS patients ranged from 0.74% to 2.49%, while the prevalence of renal failure ranged from 0% to 0.78%.^[21]^

### 4.2. Types of renal diseases associated with MS

#### 4.2.1. Nephrolithiasis.

The most common cause of kidney injury is post-renal, related to bladder dysfunction, which leads to recurrent urinary tract infections (UTI) and renal stones.^[30]^ Due to urinary stasis or the use of a catheter, people with MS are increasingly susceptible to recurrent UTI, and if left untreated, it may lead to nephrolithiasis, pyelonephritis, and urosepsis.

A study by Ganesan et al showed that the incidence of struvite stones due to urinary stasis was 8% compared to 3% in controls.^[1]^ struvite stones are associated with the formation of staghorn calculi, and given the increased risk of UTI and bacteriuria in MS patients;^[31]^ there is a possibility of an increased risk of struvite stones and formation of staghorn calculi in MS patients.^[1]^

Ganesan et al also showed that patients with MS were more likely to present with calcium phosphate stones, as was seen in 42% of patients with MS compared to 15% of controls. This may be due to the alkaline urine pH observed in patients with UTI, which favors the formation of calcium phosphate stones.^[32]^

#### 4.2.2. Glomerulonephritis.

Glomerulonephritis seen in MS patients has been attributed to immunomodulatory therapies, such as IFN-β.^[16,23,33]^ IFN-β is the first-line treatment option for patients with relapsing and remitting MS, which is known to decrease relapse rates, reduce the burden of magnetic resonance imaging disease, and possibly delay the onset of disability.^[34]^ The proposed mechanism of action of IFN-β in MS is immune modulation via its effects on T cells, decreasing immune cell trafficking across the blood-brain barrier, inducing apoptosis of auto-reactive cells, promoting T regulatory cell activity, and inducing a shift to anti-inflammatory cytokines.^[35]^ There have been reports of FSGS, minimal change disease (MCD), membranous glomerulonephritis, membranoproliferative glomerulonephritis (MPGN), and TMA, seen in patients with MS who are on IFN-β.^[16,17,23]^

The mechanism of kidney damage from IFN-β can be either direct or indirect.^[11]^ The direct effect may be mediated by impairing the charge barrier of the glomerular basement membrane (GBM) due to the interaction between its positive charge and the negatively charged GBM.^[16]^ Indirectly, interferons may directly stimulate T cells or glomerular endothelial cells to produce other cytokines that may affect the permeability of the GBM.^[16,23]^

##### 4.2.2.1. Focal segmental glomerulosclerosis.

FSGS is one of the most common renal diseases associated with the use of immunomodulatory therapies for MS treatment. FSGS is a common cause of idiopathic nephrotic syndrome in African American patients and may be the most frequent cause of nephrotic syndrome in the general population.^[17]^ FSGS may be primary or secondary to podocytopathies due to gene mutations, drugs, viral infections, or hemodynamic stress.^[36]^ Collapsing FSGS is characterized by implosive wrinkling and collapse of the GBM with hypertrophy and hyperplasia of the overlying podocytes.^[17]^

FSGS has been widely reported in patients with MS treated with IFN-β. This was discovered by Tornes et al, where an African-American obese woman with hypertension and MS developed FSGS within 4 months of treatment with IFN.^[11]^ Prior to treatment, she had normal renal function. Renal biopsy with immunofluorescence staining revealed the absence of antibodies and complement deposition; the findings in this patient ruled out an immune complex-mediated mechanism of renal damage. After discontinuation of IFN, clinical and laboratory findings showed resolution of FSGS.^[11]^ Markowitz et al reported a cohort of 11 patients treated with all types of IFN (α, β, and γ) that developed FSGS; these included 3 women who had MS, 2 of whom had comorbid hypertension, and 2 also had coexisting diabetes and obesity.^[17]^ These 3 patients were treated with IFN beta and had varying clinical presentations, including edema, proteinuria, and renal insufficiency.^[37]^ Of note, 1 patient was treated with IFN-β-1a for 4 years and developed proteinuria and renal insufficiency. IFN-β was discontinued, resulting in the normalization of renal function and a marked decline in proteinuria.^[37]^ She was then restarted on the same IFN formulation more than 3 years later, which also led to massive proteinuria (9.97 g/d) and an elevated creatinine of 1.5 mg/dL. Following discontinuation of IFN, there was a reduction in proteinuria (1.4 g/dL) and improvement in renal function with a creatinine of 1.2 mg/dL. All 3 patients had FSGS, of which 2 had resolution of symptoms following discontinuation of IFN.^[37]^

Ozturk et al also described a case of a 32-year-old male patient with MS who had been on IFN-β-1a therapy for 6 years, who had proteinuria (3 + protein) on urine dipstick test and quantitative proteinuria of 1671 mg/day, which subsequently worsened to 3 g/day a month later.^[12]^ This necessitated the cessation of IFN therapy, as no other risk factors were identified. Renal biopsy revealed FSGS; he was placed on irbesartan and proteinuria gradually resolved over a 6-month duration.^[12]^

##### 4.2.2.2. Minimal change disease.

MCD has also been associated with immunomodulatory therapy for the treatment of MS, especially with the use of interferon β. Patients presented with clinical features of nephrotic syndrome (nephrotic-range proteinuria, hypoalbuminemia, and generalized edema) following the initiation of IFN-β therapy.

Kumaska et al reported a case of a 43-year-old woman with MS and nephrotic syndrome 21 months after initiation of treatment with subcutaneous interferon (IFN)-β-1b for maintenance therapy.^[16]^ She underwent a percutaneous renal biopsy, which revealed features of MCD and was subsequently placed on oral corticosteroid 2 weeks after cessation of IFN-β. She subsequently experienced resolution of symptoms of nephrotic syndrome, and oral corticosteroids were tapered off.^[16]^

##### 4.2.2.3. Membranous glomerulonephritis.

In a study conducted by Auty and Saleh, a 28-year-old man who had MS and was placed on maintenance of IFN-β-1a was reported.^[13]^ He developed membranous glomerulonephritis after 21 months of IFN-β-1a treatment, and bilateral pedal edema extending to the thighs with presacral edema, proteinuria, and normal kidneys on renal ultrasound.^[13]^ Renal biopsy showed stage 2 membranous nephropathy with clear “spikes” visible on Jones silver stain. There was also evidence of thickened GBM and focal increase in the mesangial matrix. Following cessation of interferon therapy and treatment with corticosteroids, mycophenolate, cyclosporine, and perindopril, he showed improvement in his symptoms and attained remission of the renal disease.^[13]^

##### 4.2.2.4. Membranoproliferative glomerulonephritis.

Wallbach et al reported a case of MPGN in a 40-year-old woman who was being treated for relapsing-remitting MS using IFN-β-1b.^[14]^ She presented with features of renal dysfunction, which included massive proteinuria with protein excretion of 8.3 g/dL, and hypoalbuminemia (serum albumin of 2.9 g/dL). Her cholesterol, serum creatinine, and urea levels were within normal ranges. Blood workup showed an absence of hepatitis B and C viruses, HIV, antinuclear and antineutrophil cytoplasmic antibodies, and normal complement C3 and C4 levels. Kidney biopsy revealed features of immune complex-mediated MPGN.^[14]^ She had resolution of the renal abnormalities following replacement of IFN-β-1b with glatiramer acetate.^[14]^

#### 4.2.3. Others.

##### 4.2.3.1. Renal thrombotic microangiopathy.

TMA is a microvascular occlusive disorder characterized by the systemic or intrarenal aggregation of platelets, thrombocytopenia, and mechanical injury to erythrocytes. Although thrombotic thrombocytopenic purpura and hemolytic uremic syndrome are the two most frequently observed clinical presentations of TMA, adverse effects of drugs have been reported as a probable cause.^[15]^

Mahe et al reported a case of a 38-year-old man with MS who was treated for 5 years with IFN-β-1a who developed renal failure with hypertension due to TMA with clinical presentation of hemolytic uremic syndrome.^[15]^ Viral, bacteriological, and immunological studies, including antinuclear antibodies, were all negative. However, renal biopsy showed features of both acute and chronic TMA lesions. Biopsy findings included a few glomeruli with capillary-loop double contours, fibrin thrombi, and mesangiolysis zones. It also showed predominant chronic ischemic changes, with diffuse interstitial fibrosis, tubular atrophy, extensive ischemic changes in the glomeruli, and severe fibrous intimal thickening of the interlobular arteries. Although kidney failure persisted, there was no evidence of the recurrence in subsequent hospitalizations following the cessation of IFN-β.^[15]^ There are also other case reports of renal TMA associated with the use of IFN-β-1a in MS.^[18–20]^

##### 4.2.3.2. Acute renal failure.

There is a paucity of medical literature on acute renal failure associated with MS. The only literature reported was that of Yuruktumen et al A 64-year-old man presented with urinary retention from detrusor-sphincter dyssynergia, which is common in patients with MS.^[10]^ The patient’s symptoms and clinical findings resolved within 24 hours following urinary catheterization and administration of medications. These findings in this patient could be a result of neurogenic lower urinary tract dysfunction, which can be seen in patients with MS, and these (detrusor hyperreflexia, detrusor hypocontractility, detrusor-sphincter dyssynergia) can be diagnosed using urodynamic studies.^[10]^

### 4.3. Prognosis of renal diseases in patients with MS

The prognosis of renal disease in patients with MS differs according to the etiology, type, duration of disease, and patient factors. Most diseases, especially nephrolithiasis, glomerulonephritis induced by immunomodulatory therapies, and acute renal failure, have favorable prognosis when discovered in a timely manner and treated appropriately. However, in a few cases (such as those with renal TMA), kidney disease could persist and progress to chronic renal disease.

## 5. Conclusion

Although kidney disease can be associated with MS, few cases have been reported in the literature. The pathogenesis of renal disease in these patients can be related to lower urinary tract dysfunction, recurrent UTI, and treatment with immunomodulatory agents such as interferons. Therefore, neurologists and other physicians managing patients with MS should be aware of the risk of renal disease and monitor patients’ renal function closely during the course of treatment. Further studies are needed to explore renal diseases and their prevention in patients with MS.

## Authors contribution

**Conceptualization:** Gabriel Alugba, Alexsandra Urhi, Iyanu Victoria Olateju, Henry Onyemarin, Consolata Uzzi, Adeolu Funso Oladunjoye, Olubunmi Oladunjoye.

**Data curation:** Gabriel Alugba, Alexsandra Urhi, Iyanu Victoria Olateju, Henry Onyemarin, Hakeem Adegboyega Popoolah, Emmanuel Ukenenye, Gideon Asaolu, Adeolu Funso Oladunjoye.

**Methodology:** Gabriel Alugba, Alexsandra Urhi, Iyanu Victoria Olateju, Henry Onyemarin, Consolata Uzzi, Tolulope Oshiba-Fowode, Elvis Obomanu, Hakeem Adegboyega Popoolah, Emeka J. Okoronkwo, Emmanuel Ukenenye, Gideon Asaolu, Adeolu Funso Oladunjoye, Olubunmi Oladunjoye.

**Project administration:** Gabriel Alugba, Alexsandra Urhi, Iyanu Victoria Olateju, Henry Onyemarin, Consolata Uzzi, Tolulope Oshiba-Fowode, Elvis Obomanu, Hakeem Adegboyega Popoolah, Emmanuel Ukenenye, Gideon Asaolu, Adeolu Funso Oladunjoye, Olubunmi Oladunjoye.

**Software:** Gabriel Alugba, Alexsandra Urhi.

**Supervision:** Gabriel Alugba, Alexsandra Urhi, Consolata Uzzi, Hakeem Adegboyega Popoolah, Adeolu Funso Oladunjoye, Olubunmi Oladunjoye.

**Validation:** Gabriel Alugba, Alexsandra Urhi, Iyanu Victoria Olateju, Henry Onyemarin, Emeka J. Okoronkwo, Emmanuel Ukenenye, Adeolu Funso Oladunjoye, Olubunmi Oladunjoye.

**Visualization:** Gabriel Alugba, Alexsandra Urhi, Henry Onyemarin, Adeolu Funso Oladunjoye, Olubunmi Oladunjoye.

**Writing – original draft:** Gabriel Alugba, Alexsandra Urhi, Iyanu Victoria Olateju, Henry Onyemarin, Consolata Uzzi, Tolulope Oshiba-Fowode, Elvis Obomanu, Hakeem Adegboyega Popoolah, Emeka J. Okoronkwo, Emmanuel Ukenenye, Gideon Asaolu, Adeolu Funso Oladunjoye, Olubunmi Oladunjoye.

**Writing – review & editing:** Gabriel Alugba, Alexsandra Urhi, Iyanu Victoria Olateju, Henry Onyemarin, Consolata Uzzi, Tolulope Oshiba-Fowode, Elvis Obomanu, Hakeem Adegboyega Popoolah, Emeka J. Okoronkwo, Emmanuel Ukenenye, Gideon Asaolu, Adeolu Funso Oladunjoye, Olubunmi Oladunjoye.
